# A novel FOXO1-mediated dedifferentiation blocking role for DKK3 in adrenocortical carcinogenesis

**DOI:** 10.1186/s12885-017-3152-5

**Published:** 2017-03-01

**Authors:** Joyce Y. Cheng, Taylor C. Brown, Timothy D. Murtha, Adam Stenman, C. Christofer Juhlin, Catharina Larsson, James M. Healy, Manju L. Prasad, Wolfram T. Knoefel, Andreas Krieg, Ute I. Scholl, Reju Korah, Tobias Carling

**Affiliations:** 1 Department of Surgery & Yale Endocrine Neoplasia Laboratory, Yale University School of Medicine, New Haven, CT USA; 2 Department of Pathology, Yale University School of Medicine, New Haven, CT USA; 3 Department of Oncology-Pathology, Karolinska Institutet, Karolinska University Hospital, CCK, Stockholm, Sweden; 4 Department of Surgery, Medical School, Heinrich Heine University, University Hospital Düsseldorf, Düsseldorf, Germany; 5 Department of Nephrology, Medical School, Heinrich Heine University, University Hospital Düsseldorf, Düsseldorf, Germany; 6 Department of Surgery, Yale University School of Medicine, 333 Cedar Street, FMB130A, New Haven, CT 06520 USA

**Keywords:** DKK3, FOXO1, Adrenocortical carcinogenesis

## Abstract

**Background:**

Dysregulated WNT signaling dominates adrenocortical malignancies. This study investigates whether silencing of the WNT negative regulator DKK3 (Dickkopf-related protein 3), an implicated adrenocortical differentiation marker and an established tumor suppressor in multiple cancers, allows dedifferentiation of the adrenal cortex.

**Methods:**

We analyzed the expression and regulation of DKK3 in human adrenocortical carcinoma (ACC) by qRT-PCR, immunofluorescence, promoter methylation assay, and copy number analysis. We also conducted functional studies on ACC cell lines, NCI-H295R and SW-13, using siRNAs and enforced DKK3 expression to test DKK3’s role in blocking dedifferentiation of adrenal cortex.

**Results:**

While robust expression was observed in normal adrenal cortex, DKK3 was down-regulated in the majority (>75%) of adrenocortical carcinomas (ACC) tested. Both genetic (gene copy loss) and epigenetic (promoter methylation) events were found to play significant roles in DKK3 down-regulation in ACCs. While NCI-H295R cells harboring β-catenin activating mutations failed to respond to DKK3 silencing, SW-13 cells showed increased motility and reduced clonal growth. Conversely, exogenously added DKK3 also increased motility of SW-13 cells without influencing their growth. Enforced over-expression of DKK3 in SW-13 cells resulted in slower cell growth by an extension of G1 phase, promoted survival of microcolonies, and resulted in significant impairment of migratory and invasive behaviors, largely attributable to modified cell adhesions and adhesion kinetics. DKK3-over-expressing cells also showed increased expression of Forkhead Box Protein O1 (FOXO1) transcription factor, RNAi silencing of which partially restored the migratory proficiency of cells without interfering with their viability.

**Conclusions:**

DKK3 suppression observed in ACCs and the effects of manipulation of DKK3 expression in ACC cell lines suggest a FOXO1-mediated differentiation-promoting role for DKK3 in the adrenal cortex, silencing of which may allow adrenocortical dedifferentiation and malignancy.

**Electronic supplementary material:**

The online version of this article (doi:10.1186/s12885-017-3152-5) contains supplementary material, which is available to authorized users.

## Background

Adrenocortical carcinoma (ACC) is a rare (0.5–2 cases per million/year) endocrine malignancy that carries a poor prognosis at diagnosis due to its propensity to metastasize before detection. Even with aggressive surgical and oncologic therapy, the 5-year survival rate is an abysmal 16–38% [1–4]. A major reason for the lack of an effective targeted treatment strategy for ACCs is an inadequate understanding of the molecular pathogenesis of the disease [3, 4].

Genetic and epigenetic dysregulations of the WNT, p53, and IGF2 pathways appear to dominate various cancer-driving anomalies in the majority of ACCs [5–7]. Recent findings from comprehensive genetic analyses of ACCs confirmed a signature role for WNT dysregulation in the origin and/or progression of ACCs [4, 6, 8, 9]. Physiologically, both canonical and non-canonical WNT signaling pathways play global and zone-specific roles in the development, differentiation, and homeostasis of the adrenal gland [10, 11]. In particular, endocrine homeostasis of the adrenal glomerulosa and fasciculata zones is largely controlled by WNT-differentiation signaling mediated by the Wnt4-Fz1/2-Dvl3-β-Catenin-SF1 axis [12–16]. Regulatory components of this proposed adrenal cortex-specific Wnt4 axis include the secretory factors, frizzled-related protein 1 (SFRP1) and the putative tumor suppressor, DKK3 [14, 17, 18]. Aberrant WNT signaling has been well-established in the origin of many tumor types and is strongly associated with stabilization of β-catenin in the cytoplasm and/or in the nucleus and constitutive activation of WNT target genes [19, 20]. Similar stabilization and nuclear accumulation of β-catenin is seen in benign adrenocortical adenomas (ACAs) and frequently in malignant ACCs [10, 21]. However, only 10% of ACCs with constitutively active β-catenin carry mutations in the β-catenin gene (*CTNNB1*), suggesting alternate mechanisms of aberrant WNT activation, including dysregulation of WNT inhibitors such as Wif-1 [22]. Other WNT regulatory mutations found in ACCs include *PRKAR1A* [23] and recently identified *KREMEN1* and *ZNRF3* gene deletions [8, 24].

Although implicated in zonal differentiation and hormone biosynthesis [14, 25], a definitive role for the ubiquitous WNT inhibitor DKK3 in promoting functional differentiation and/or blocking tumor dedifferentiation of the adrenal cortex has yet to be clarified. The inhibitory role of DKK3 in WNT signaling is context-dependent and appears to be influenced by a repertoire of cell surface receptors and co-expressed ligands [26]. DKK3, a 38 kDa secreted glycoprotein with an N-terminal signal peptide, is also implicated in eliciting distinct intracellular roles in addition to its secretory functions [27]. Reduced DKK3 expression is observed in a variety of solid tumors, and re-expression studies in multiple cancer cell types mostly resulted in cell cycle arrest and/or apoptosis, strongly suggesting a global tumor suppressor role for this WNT regulator (reviewed in [26]). Furthermore, ectopic expression of DKK3 in a variety of cancer cell types stifled aggressive malignant behavior, reversed epithelial-mesenchymal transition (EMT), and impaired cell motility, pointing towards a comprehensive dedifferentiation-blocking role for DKK3 [28, 29]. This study investigates a potential tumor suppressor role for the implicated adrenal differentiation factor DKK3 in blocking dedifferentiation of adrenocortical cells.

## Methods

### Tissue acquisition

Written informed consent was obtained from patients prior to surgical resection of adrenal tissue according to protocols approved by Institutional Review Boards at (a) Yale University, New Haven, CT, USA, (b) Heinrich Heine University Düsseldorf, Düsseldorf, Germany, and (c) Karolinska Institutet, Stockholm, Sweden. Tissue samples were flash-frozen (FF) in liquid nitrogen and stored at −80 °C until processed for study. Specimens displaying unequivocal histopathological characteristics of ACCs (*n* = 38) and histologically normal adrenal tissue (*n* = 14) samples excised with ACAs were selected for study. Consecutive unstained/hematoxylin & eosin (H&E) stained 5 μM sections of formalin-fixed, paraffin-embedded (FFPE) tissue samples underwent immunohistochemistry analyses. All samples were histopathologically confirmed by experienced endocrine pathologists before processing.

### DNA, RNA, and protein preparation

Genomic DNA and total RNA were isolated from FF samples using AllPrep DNA/RNA/Protein Mini Kit (Qiagen) as per manufacturer’s recommendations. Quantity and quality of prepared nucleic acids were assessed by spectrophotometry (NanoDrop Technologies, Inc.). Total protein from cultured cells was extracted using Laemmli buffer (BioRad) as cell lysis buffer; protein concentrations were quantified using Pierce BCA Protein Assay Kit (ThermoFisher Scientific) and GloMax multidetection system (Promega), as per manufacturer’s instructions.

### Gene expression analysis

Total RNA (100 ng) was reverse transcribed using iScript cDNA synthesis kit (Bio-Rad) as per manufacturer’s instructions. Quantitative real-time PCR (qRT-PCR) was performed in triplicate using TaqMan PCR master mix with FAM fluorophore and probe/primer pairs specific to human *DKK3* (Hs00951307_m1), *FOXO1* (Hs01054576_m1), and *RPLP0* (Hs99999902_m1) (ThermoFisher Scientific) according to manufacturer’s cycling conditions using CFX96 thermal cyclers (Bio-Rad). Gene expression levels were normalized to mean *RPLP0* expression levels. Relative gene expression values were calculated using recommended Livak method (Bio-Rad). Fold-change expression values were calculated by base-two logarithmic transformations of relative gene expression values.

For pathway-focused gene expression analysis, (a) RT^2^ Profile PCR Array Human WNT Signaling Pathway and (b) RT^2^ Profiler PCR Array Human Transcription Factors were used according to protocol outlined in RT^2^ Profiler PCR Array Handbook (Qiagen). Briefly, 100 ng of DNA-free RNA from each sample was used for 84 target genes listed in gene lists (available at www.qiagen.com) using 96-well RT^2^ profiler array format D. cDNA was prepared using RT^2^ first strand kit and amplified using RT^2^ SYBR Green Mastermix (both from Qiagen) using CFX96 thermal cycler. Differential expression of target genes was calculated using ∆∆C_T_ method on data web portal at www.SABiosciences.com/pcrarraydataanalysis.php.

### Methylation-specific PCR

Methylation status of CpG island A of *DKK3* promoter (Chr11:12029737–12030841) was assessed by MethylScreen technology using EpiTect Methyl II PCR Assay (Qiagen) as previously described [30]. Briefly, 125 ng of genomic DNA was mock-digested or digested with methylation-sensitive and methylation-dependent restriction enzymes individually or together, and methylation status of target DNA sequence was measured using qRT-PCR with probes specific to target *DKK3* promoter sequence. C_T_ values were converted into percentages of unmethylated, intermediate-methylated, and hypermethylated CpG values using a quantitation algorithm from EpiTect Methyl II PCR Assay Handbook (Qiagen). Tissue samples were designated as hypermethylated (>5% alleles with hypermethylation), intermediate-methylated (>5% alleles with intermediate methylation), or unmethylated (no methylation detected).

### DNA copy number analysis (CNA) by qRT-PCR

DNA from 27 ACC samples that passed specified test quality criteria were analyzed in quadruplicate with TaqMan Copy Number Assay using a primer / probe pair specific to target gene *DKK3* or housekeeping gene *RPPH1*. Normal adrenal tissue was used for diploid (2n) reference. Copy numbers were predicted using CopyCaller software v2.0 (ThermoFisher Scientific). TaqMan Copy Number Assay used was Hs00228043_cn. Target gene *DKK3* located on Chr.11:11989984 on NCBI build 37. Housekeeping gene Ribonuclease P RNA Component H1, *RPPH1* located on Chr.14:20811565 on NCBI build 37.

### Immunofluorescence (IF) detection of proteins

Five μM-thick FFPE sections were processed for immunofluorescence detection of DKK3 and β-catenin proteins as described previously [31]. Goat anti-DKK3 polyclonal (SC14959; 1:100 dilution) or mouse anti-β catenin monoclonal (SC47778; 1:200 dilution) primary antibodies and anti-goat FITC (fluorescein isothiocyanate) and anti-mouse TR (Texas Red) secondary antibodies (1:1000) were used, followed by Ultracruz mounting agent containing 4′,6-diamidino-2-phenylindole (DAPI) (all from Santa Cruz Biotechnology, Inc.) for indirect immunodetection. A Zeiss AX10 confocal microscope with AxioVision 4.8 program was used for IF analysis, and photomicrographs were taken at a total magnification of 100× or 400×, as noted.

### Cell culture, expression vectors, transfections, and western blot detection

American Type Culture Collection (ATCC)-authenticated human ACC cell lines SW-13 (CCL-105) and NCI-H295R (CRL-2128) were maintained in growth conditions recommended by ATCC, as reported previously [31]. For DKK3 treatments, a working concentration of 5 μg/mL (in PBS) of human recombinant DKK3 (R&D Systems) was used. RNAi silencing was carried out with 3 unique 27-mer siRNA duplexes (designated siA, siB, and siC) targeting *DKK3* (Human) and *FOXO1* (Human) transcripts. Universal scrambled negative control siRNA was used as non-specific control (all from Origene). Lipofectamine2000-mediated transfection was carried out in Opti-MEM according to manufacturer’s recommendations (ThermoFisher Scientific) in 6-well plates with starting densities of 50,000 cells/well for SW-13 and 80,000 cells/well for NCI-H295R. Transfection medium was replaced with regular growth medium after 24 h of transfection. Cells were lysed for RNA extraction (after 24 h) or protein extraction (after 48 h), and assays were done 48 h post-transfection.

Myc-DDK tagged pCMV6-Entry, pCMV6-Entry/GFP, and pCMV6-Entry/*DKK3* plasmid vectors (Origene) were used for transient and stable expression. Transient transfection was carried out in Opti-MEM medium using Lipofectamine2000 according to manufacturer’s recommendations (ThermoFisher Scientific) in 6-well plates with starting densities of 50,000 cells/well for SW-13 and 80,000 cells/well for NCI-H295R cells. Cells were transfected one day after plating. Transfection medium was replaced with appropriate growth medium 6 h post-transfection, and cells were assayed for cell behaviors 24 h post-transfection. Total cell numbers and viability were calculated by staining cells with 0.4% Trypan Blue (ThermoFisher Scientific) and counting with hemocytometer (Hausser Scientific Co.). Experiments were performed in triplicate, and parallel pCMV6-Entry/GFP transfections were used to determine transfection efficiency.

Stable Geneticin (G418)-resistant pCMV6-Entry, pCMV6-Entry/GFP, and pCMV6-Entry/DKK3 transfected clones were selected in 800 μg/mL G418-containing growth medium (ThermoFisher Scientific). Multiple clones were then pooled into populations to avoid expression variability and selection bias between clones. Established populations designated SW-Neo (from pCMV6-Entry transfections) and SW-DKK3 (expressing Myc-DDK/DKK3) were compared to parental SW-13 cells to determine effects of constitutive DKK3 expression on SW-13 cells’ malignant properties. Constitutive DKK3 expression was confirmed via qRT-PCR using TaqMan primer/probe pairs (ThermoFisher Scientific) and Western blotting using anti-DKK3 mAb (1:500; Abcam), anti-mouse-HRP (Santa Cruz Biotechnologies, Inc.), Mini-PROTEAN TGX gel, PVDF blotting membrane (Bio-Rad), and enhanced chemiluminescence (ECL) detection reagents (ThermoFisher Scientific) as per manufacturer’s protocols. Unless specified, 100 μg protein was loaded per well of 4–10% SDS gels (Bio-Rad). Equal protein loading was confirmed by staining PVDF membranes with GelCode Blue Safe Protein stain (ThermoFisher Scientific) after chemiluminescence detection.

### Flow cytometric analysis of cell cycle

SW-13, SW-Neo, and SW-DKK3 cells were fixed in cold 70% ethanol for 30 min at 4 °C, washed twice with PBS, treated with ribonuclease (100 μg/mL), and stained with propidium iodide (PI; 50 μg/mL in PBS). Using bandpass filter 605 nm (for PI), forward and side scatter were measured in a BD LSRII Flowcytometer. Pulse processing was used to exclude cell doublets from the analysis. FlowJo software was used to analyze the best Gaussian distribution curve to each peak for the cell populations of G0-G1 and G2-M.

### Cell invasion, migration, adhesion, and clonogenic growth assays

To assess invasive proficiencies, 100,000 SW-13, SW-Neo, or SW-DKK3 cells were allowed to invade through Matrigel from upper chambers containing serum-free medium to lower chambers containing 10% FBS medium in BD BioCoat Matrigel invasion chambers (BD Biosciences). After 24 h, Matrigel was removed, and invaded cells were fixed in 3.7% formaldehyde/PBS (10 min), stained with 0.05% crystal violet (30 min), and counted at 100X magnification with light microscope. Matrigel invasion assay was performed twice in triplicate chambers. In migration assays, 100,000 cells were allowed to migrate through 8 μM-pore size modified Boyden Chambers (BD Biosciences) from upper chambers containing serum-free medium to lower chamber with 10% FBS medium. After 4 or 8 h, cells that migrated to lower side of the membrane were fixed, stained, and counted as above.

Cell adhesion assays were carried out in 6-well plates. One hundred thousand cells were seeded per well, allowed to grow overnight, washed with warm PBS, and incubated with 0.5 mL of 0.25% Trypsin-EDTA for 1 min; Trypsin-EDTA was then removed, plates were tapped gently to remove loosely attached cells, cells were washed with 10% FBS medium, fixed, stained and counted as above. For clonogenic growth assays, cells were seeded in 6-well plates at low densities (5,000 cells/well) and allowed to grow 7 days in appropriate growth medium (SW-Neo and SW-DKK3) with medium change every 3 days. On day 7, cells were washed with PBS, fixed, and stained as above. Colonies with 12 ± 2 or 4 ± 2 cells were counted as separate groups and averaged from 6 wells. Experiments were repeated 3 times, and data from a representative experiment is presented.

### Statistical analysis

Normal distribution of continuous variables was assessed using D’Agostino and Pearson omnibus tests. Normally distributed variables were analyzed using 2-tailed *t* test; Mann–Whitney *U* test was used for non-normally distributed variables. For variables with greater than 2 dependent values, a 1-way analysis of variance and Kruskal-Wallis tests were used for normally and non-normally distributed populations, respectively. Matched continuous variables were compared using Pearson correlation. Survival data were assessed by Kaplan-Meier methods, and differences were compared by Mantel-Cox test. Statistical analyses were performed using Prism 6 (GraphPad Software).‬‬‬

## Results

### Reduced expression of *DKK3* in adrenocortical carcinoma

**Fig. 1:**
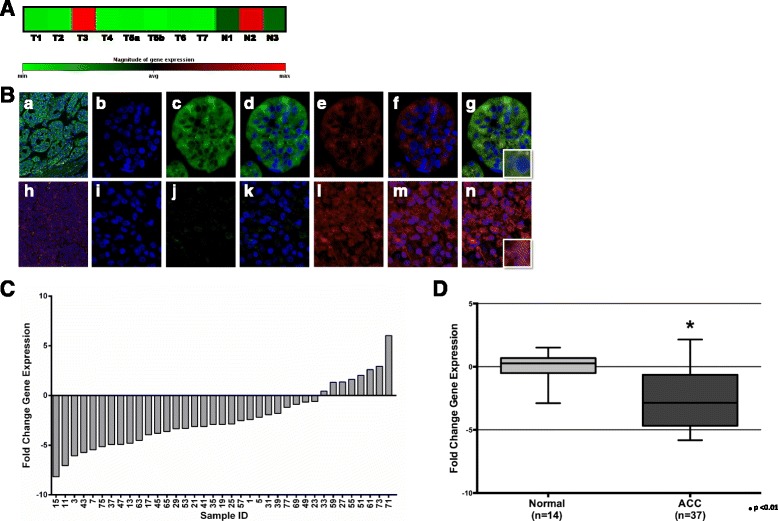
Reduced DKK3 expression in ACC. **a** Reduced DKK3 gene expression in 7 tumor samples (T1–T7) compared to 3 histologically normal adrenal samples (N1–N3). T5a and T5b: RNA from two different areas of one tumor. Magnitude of gene expression relative to housekeeping gene panel shown below. **b** Immunofluorescence detection of DKK3 and β-catenin in normal adrenal cortex (*a-g*) and ACC (*h-n*). Tissue sections treated with primary/secondary antibodies for DKK3 (FITC, *green*; *c, j*) or β-catenin (TR, *red*; *e*, *l*), DAPI (*blue* for nuclear staining; *b*, *i*), or combinations of FITC/DAPI (*d*, *k*), TR/DAPI (*f*, *m*), or FITC/TR/DAPI (*a*, *h*, *g*, *n*). *a* and *h*: 100× magnification; *b*-*g*, *i*-*n*: 400× magnification; inset (*g*, *n*): 1000× magnification. **c** DKK3 gene expression (fold-change) in 37 ACC samples relative to average expression of 14 normal adrenal samples normalized to 1. **d** Average DKK3 expression (fold-change) in study cohort (*n* = 37) compared to average expression from 14 normal adrenal samples

Recent comprehensive genetic analyses identified WNT signaling as the most common target of genetic aberrations in ACCs. To identify novel WNT targets, we compared the expression pattern of selected positive and negative WNT regulators in 7 ACC samples using an expanded WNT expression array. Among various differentially expressed WNT regulators, the expression of DKK3, a negative WNT regulator and a putative tumor suppressor in a wide variety of tumors, was found significantly reduced in the majority (6/7) of the ACC samples tested (Fig. 1a; Additional file 1: Figure S1). Further, compared to the robust expression pattern in adrenal cortex, DKK3 protein expression was found to be nearly absent in ACCs by indirect immunofluorescence analysis (Fig. 1b; a&h). DKK3 was observed to be expressed in the zona fasciculata and zona reticularis (data not shown) in normal adrenal cortex, though to a lesser extent going inward from the zona glomerulosa (Fig. 1b). In contrast to the near absence of DKK3, β-catenin appeared to be over-expressed in ACC (Fig. 1b; h). Moreover, both robustly expressed DKK3 and weakly expressed β-catenin proteins were found predominantly in the cytoplasmic compartment of normal adrenal cortex (Fig. 1b; b-g), while increased β-catenin levels were found both in the cytoplasm and the nuclei of ACC cells (Fig. 1b; i-n). Due to the rarity of the disease and scarcity of fresh-frozen samples, an international patient cohort (*n* = 38) was assembled for DKK3 expression analysis (Table 1). Quantitative RT-PCR analysis of 37 ACC samples confirmed reduced mRNA expression in the majority (70%; 26/37) of ACC samples (Fig. 1c). The mean expression of DKK3 in 37 ACCs was significantly decreased (*p* = 0.002) compared to mean DKK3 expression in 14 normal adrenal tissue samples (Fig. 1d). The high frequency of DKK3 silencing (70%) observed in ACCs is very similar to that observed in other malignancies including thyroid [32] and pancreatic cancers [33].

**Table 1:** Summary of cohort demographics and patient characteristics

Characteristics	Number of Cases	Percentage
Total Number	38	NA
Gender		
Male	14	35.8%
Female	24	63.2%
Age ± SD (y)	57.7 ± 13.2	NA
Cohort		
Yale	10	26.3%
Karolinska	25	65.8%
Düsseldorf	3	7.9%
Tumor Size (cm)		
Mean ± SD	12.8 ± 4.4	NA
Range	5.5–21.0	NA
ENSAT 2008 Stage		
I	0	0.0%
II	18	47.4%
III	11	28.9%
IV	9	23.7%
Hormone Hypersecretion		
Aldosterone	1	2.6%
Cortisol	9	23.7%
Androgen/DHEA	4	10.5%
Estrogen	1	2.6%
Multi-secreting^a^	5	13.2%
Non-functional	14	36.8%
No information available	4	10.5%
Outcome		
Alive, no recurrence	11	28.9%
Alive, recurrence	5	13.2%
Death from disease	16	42.1%
Death from other causes	4	10.5%
Lost to follow-up	2	5.3%

^a^Tumors secreting two or more of the following hormones: aldosterone, cortisol, testosterone, or DHEA

*y* years, *cm* centimeter, *SD* standard deviation, *ENSAT* European Network for the Study of Adrenal Tumors, *DHEA* dehydroepiandrosterone, *NA* not applicable

To determine whether reduced DKK3 expression correlated with disease presentation and/or outcome, we analyzed statistical correlation to various patient characteristics (Table 1), including age, gender, tumor size, tumor weight, ENSAT stage, and hormone secretion phenotypes. Despite the limited cohort size (*n* = 38), reduced DKK3 expression showed a non-significant trend (*p* = 0.062) towards female gender (Additional file 1: Figure S2). Kaplan-Meier survival analysis also did not reveal a significant effect on survival in patients with reduced DKK3 expression (*p* = 0.19) (Additional file 1: Figure S3).

### *DKK3* promoter methylation and gene copy number alterations in ACC

Promoter methylation has been identified as the principal mechanism of DKK3 silencing in multiple tumor types [34–39]. Moreover, we have previously shown potential involvement of global and gene-specific promoter methylation changes in ACCs [40]. Using the EpiTect protocol [31], we analyzed methylation status of the *DKK3* promoter in 9 normal adrenal tissue and 29 ACC samples. Compared to the *DKK3* promoter methylation status in normal adrenal DNA, 4 ACC samples (14%) showed marked levels of hypermethylation, and 14 samples (48%) showed intermediate-range methylation (Table 2). Twelve of 18 ACC samples with hyper- or intermediate promoter methylation (67%) also showed significant reduction in DKK3 expression, concurring with the established role of promoter methylation in *DKK3* silencing in other tumors [36, 37]. Interestingly, 8/11 samples with non-methylated promoters also showed comparable frequency of *DKK3* silencing (72%), suggesting alternate mechanisms for DKK3 down-regulation in ACC.

**Table 2:** *DKK3* mRNA expression, promoter methylation, and gene copy number alterations in adrenocortical carcinoma

Sample	Gene Expression	Promoter Methylation	Gene Copy Number
1	L	IM	2
3	L	HM	2
5	L	HM	2
7	L	UM	1
11	L	UM	1
13	L	UM	2
15	L	UM	ND
17	L	UM	1
19	L	HM	1
21	L	IM	2
25	L	IM	2
29	L	IM	1
31	L	IM	2
35	L	ND	ND
37	L	ND	ND
39	L	ND	ND
41	L	IM	2
43	L	ND	ND
45	L	UM	ND
47	L	IM	1
53	L	IM	1
57	L	HM	6
63	L	UM	2
65	L	UM	3
75	L	ND	ND
77	L	ND	ND
9	ND	IM	2
23	N	UM	2
33	N	IM	2
49	N	IM	2
71	N	ND	ND
27	H	IM	1
51	H	IM	3
55	H	IM	1
59	H	UM	2
61	H	UM	2
69	H	ND	ND
73	H	ND	ND

*Abbreviations*: *DKK3* Dickkopf-related protein 3, *L* low expression, *N* normal expression, *H* high expression, *UM* unmethylated, *IM* intermediate methylation, *HM* hypermethylation, *ND* not determined

Recent genetic analyses of ACCs by us and others have shown significant copy number alterations in genes potentially involved in various signaling pathways [30]. To determine if gene copy loss contributed to reduced expression of DKK3 in this cohort of ACC samples, we analyzed copy number variations using the TaqMan copy number assay. We found copy losses in 9 samples (33%) and copy gains in 3 of 27 ACC samples tested (Table 2; Additional file 1: Figure S4). Seven of the 9 samples with copy loss (78%) showed marked reduction in DKK3 expression; 4 showed concurrent *DKK3* promoter methylation. Interestingly, one ACC sample (ID #57) with 6 copies of the *DKK3* gene also showed promoter hypermethylation and reduced expression of DKK3.

### DKK3 silencing reduces clonogenic growth and promotes migration of ACC cells

**Fig. 2:**
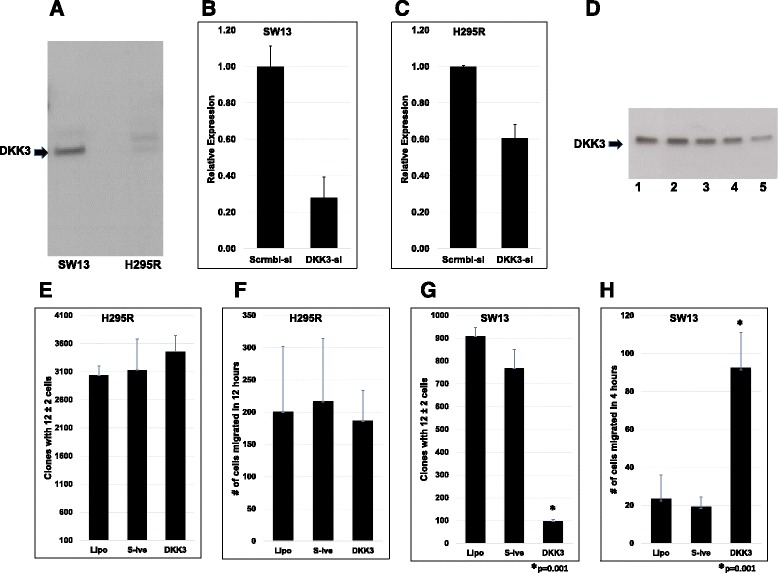
RNAi silencing of DKK3 in ACC cell lines and effects on cell behavior. **a** Western immunoblot detection of endogenous DKK3 in SW-13 (*left*) and NCI-H295R (*right*). **b** and **c**, Relative expression of DKK3 as determined by qRT-PCR in siRNA-treated SW-13 (2B) and NCI-H295R (2C) cells, normalized to expression in cells treated with scrambled siRNA for 24 h. **d** Western immunoblot detection of DKK3 in SW-13 cells treated with control (1), scrambled negative siRNA (2), 10 (3), 20 (4), and 40 nM (5) DKK3 siRNAs for 24 h followed by protein extraction 48 h post-transfection. **e-h**, NCI-H295R (**e** and **f**) or SW-13 (**g** and **h**) cells treated with Lipofectamine (Lipo), scrambled negative siRNA (S-ive), or DKK3 siRNA (DKK3) for 24 h, allowed to grow in clonogenic growth conditions (**e** and **g**), or allowed to migrate through modified Boyden chambers through growth factor concentration gradient for 12 (**f**) or 4 (**h**) hours. Clones with 12 ± 2 cells were fixed, stained, and counted with light microscope (**e** and **g**); cells that migrated to lower side of modified Boyden chamber membranes were fixed, stained, and counted (**f** and **h**)

To test whether DKK3 plays a tumor suppressor role in ACC in vitro, we investigated the expression pattern and regulation of *DKK3* in two ACC cell lines, SW-13 and NCI-H295R. Western blot analysis showed modest expression of DKK3 in SW13 cells, while NCI-H295R cells showed low expression (Fig. 2a). Despite carrying *TP53* gene mutations, non-hormone-secreting SW-13 cells maintain an unperturbed and modifiable WNT signaling pathway, whereas the adrenal hormone-producing NCI-H295R cells harbor *CTNNB1* and *axin1* mutations, resulting in constitutive WNT activation [31, 41]. To test whether suppressing endogenous DKK3 will influence malignant properties of ACC cells, we used a transient siRNA-silencing method. Silencing of *DKK3* expression in SW-13 (Fig. 2b, d) and NCI-H295R (Fig. 2c) cells with siRNA was confirmed by qRT-PCR (Fig. 2b-c) and Western blot (Fig. 2d). DKK3 silencing did not result in significant loss of viability in either cell type for the duration of study (48 h). Due to low baseline levels of DKK3 in H295R (Fig. 2a), siRNA-mediated silencing has no detectable effect observable by Western (data not shown). Next, we examined whether silencing of DKK3 modulates clonal growth or migratory potential of ACC cells. Partial silencing (40% suppression; Fig. 2c) of DKK3 (Fig. 2a) did not appear to influence clonogenic growth or migratory potentials of NCI-H295R cells (Fig. 2e-f). It is conceivable that the constitutively active WNT signaling in these cells may have conferred inherent resistance to DKK3 signaling. On the other hand, DKK3 silencing in SW-13 cells (75% suppression; Fig. 2b; lane 5 of Fig. 2d) significantly impaired the cells’ ability to form colonies in isolation (*p* = 0.001) (Fig. 2g) and promoted their motility behavior (*p* = 0.001) (Fig. 2h; Additional file 1: Figure S7). These results suggest a potential role for DKK3 silencing in adrenal carcinogenesis, which could be overrun by gain-of-function WNT mutations.

### Exogenous DKK3 promotes migration of SW-13 cells

**Fig. 3:**
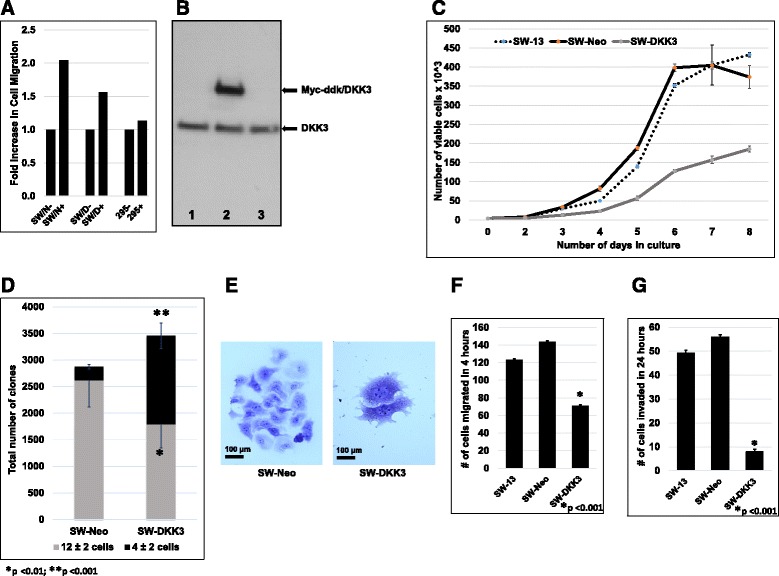
ACC cells were either treated with exogenous recombinant DKK3 (**a**) or enforced to express Myc-DDK tagged DKK3 (**b**) and assayed for cell behaviors. **a** SW-13 N/D (*left*) or NCI-H295R (*right*) cells were untreated (SW N/D-, 295-) or treated (SW N/D+, 295+) with exogenous DKK3 for 24 h and allowed to migrate through modified Boyden chamber for 4 h. Cells migrating to lower surface were fixed, stained, and counted. **b** Western immunoblot detection of endogenous DKK3 and ectopically expressed DKK3 (Myc-DDK/DKK3) in vector control (*lane 1*), SW-DKK3 (*lane 2*), or Myc-DDK/GFP control (*lane 3*) cells. **c** SW-13, SW-Neo, and SW-DKK3 cells plated in 24-well plates (5000 cells/well) were grown 8 days. Quadruplicate wells from each cell type were trypsinized, incubated in 0.2% Trypan *blue*, and viable cells were counted using hemocytometer. Data shown represent one of three independent experiments. **d** and **e**, Five thousand SW-13 or SW-DKK3 cells plated in 6-well plates were allowed to grow 7 days; clones were fixed, stained, and enumerated into 2 classes of (**a**) 12 ± 2 cells (*filled light grey*) and (b) 4 ± 2 cells (*filled black*). Majority of clones formed from SW-Neo cells were large (**e**; *left*), while SW-DKK3 cells produced a significant number of small colonies (4 ± 2) comprised of large cells (**e**; *right*). **f** One hundred thousand SW-13, SW-Neo, and SW-DKK3 cells were allowed to migrate through modified Boyden chamber for 4 h; cells that migrated to the lower side of the membrane were fixed, stained, and counted. **g** One hundred thousand SW-13, SW-Neo, and SW-DKK3 cells were allowed to invade through Matrigel in modified Boyden chambers for 24 h. Cells that invaded through Matrigel and migrated to the lower side of the membrane were fixed, stained, and counted

Reports suggesting distinct roles for endogenous and secreted DKK3s in cell behavior [17, 42] prompted us to test the effect of exogenous DKK3 addition to ACC cells. Cells grown in the presence of exogenous human recombinant DKK3 did not show a difference in their overall growth potentials (Additional file 1: Figure S5). However, migratory potential of SW-13 cells was found to be accentuated with exogenous DKK3 (Fig. 3a). The exogenous DKK3 in this instance appears to have a dominant effect over the motility-impeding effect of endogenous DKK3 (Fig. 3a & f). NCI-H295R cells with constitutively active β-catenin appeared to be resistant (Fig. 3a) to the exogenous DKK3-induced migration-promoting effects on SW-13 cells.

### Constitutive over-expression of DKK3 stifles malignant behavior of ACC cells

DKK3 is constitutively expressed and persistently present during zonal differentiation of adrenal cortex [14]. To test whether constitutive over-expression of DKK3 promotes redifferentiation of ACC cells, we generated a stable population of SW-13 cells engineered to over-express DKK3. Since NCI-H295R cells exhibited no appreciable response to either endogenous or exogenous DKK3 (Figs. 2e-f and 3a), we limited our attention to SW-13 cells. Expression of ectopic DKK3 was confirmed (Fig. 3b), and SW-DKK3 cells were assessed for various malignant properties compared to parental SW-13 and control SW-Neo cells.

SW-DKK3 cells grew at a slower rate compared to both parental SW-13 and SW-Neo cells (Fig. 3c). The slow rate of growth of SW-DKK3 cells was found to be caused by an increase in the percentage of cells accumulated in G1 phase (47.5% SW-Neo compared to 56.3% SW-DKK3 cells) of the cell cycle (Additional file 1: Figure S6). Since suppression of endogenous DKK3 expression resulted in reduced clonogenic growth and increased motility of SW-13 cells, we compared clonal growth and migratory potential of SW-DKK3 cells to that of SW-Neo cells, using parental SW-13 cells as reference. Compared to their vector-transfected controls, SW-DKK3 cells showed an overall increase in clonal growth efficiency (Fig. 3d). Interestingly, 52% of the clones were small (4 ± 2 cells) and composed of larger, slow-growing, or growth-arrested cells (Fig. 3e; right). In SW-Neo, this fraction of small clones represented only 9% of the clones (*p* < 0.001), while the remaining 91% constituted large colonies comprised of 12 ± 2 cells (Fig. 3e; left).

Next, we assessed the effect of constitutive DKK3 over-expression on migratory potential of SW-13 cells. SW-DKK3 cells exhibited significantly decreased migratory potential compared to parental SW13 and SW-Neo cells (*p* < 0.001) (Fig. 3f). To test whether DKK3-promoted reduction in SW-13 cells’ migratory potential has a potential in vivo implication, we performed an in vitro invasion assay. As reported previously in other cancer types [26], over-expression of DKK3 significantly impaired SW-13 cells’ ability to invade through reconstituted matrix (*p* < 0.001) (Fig. 3g).

### DKK3 promotes a more differentiated phenotype in ACC cells

**Fig. 4:**
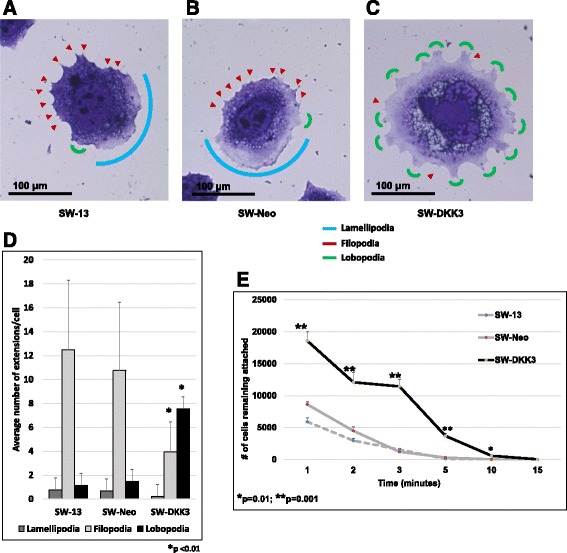
Constitutive over-expression of DKK3 reorganizes cellular extensions and cell spreading. **a**-**c** SW-13 (**a**), SW-Neo (**b**), and SW-DKK3 (**c**) cells were grown on glass cover-slips, fixed, stained, and photographed. SW-13 and SW-Neo cells show a predominance of filopodia (*red arrowheads*) around edges; SW-DKK3 shows more lobopodia (*small green arcs*), absence of lamellipodia (*blue arc*), and few filopodia around edges. While cells in **a** and **b** appear to be polarized with filopodia at leading edge and lamellipodia at lagging edge, SW-DKK3 cells (**c**) show evenly spread flat lobopodia with extensive spreading and absence of polarity. Photomicrographs are taken using light microscope at 400× magnification. **d** Average number of lamellipodia, filopodia, and lobopodia per cell calculated from manual counting of cell extensions. Twenty randomly taken (400× magnification) photomicrographs of SW-13, SW-Neo, and SW-DKK cells used for quantification. **e** One hundred thousand SW-13, SW-Neo, and SW-DKK3 cells/well of 6-well plates were allowed to grow overnight, detached at specified times, cells remaining attached were fixed, stained, and counted manually

To test whether decreased invasive behavior of DKK3-over-expressing SW-DKK3 cells is due to signaling changes that can potentially modulate cell spreading and thereby migration kinetics, cell morphology was observed under light microscopy. SW-DKK3 cells appeared to be larger with an extensive spreading phenotype aided by dysregulated cell edge attachments (Fig. 4a-c). While the parental SW-13 and SW-Neo cells displayed a significantly higher number of filopodia in a planar orientation, SW-DKK3 cells displayed a significantly higher proportion of lobopodial extensions (*p* < 0.01) (Fig. 4a-d). To test whether the differential expression of cell extensions alters cell attachment characteristics, we performed a cell-detachment assay. SW-DKK3 cells showed a significantly stronger attachment to substratum compared to both SW-13 and SW-Neo cells (*p* < 0.01) (Fig. 4e). Whether increased attachment strength to substratum or multidirectional polarity conferred by the multitude of lobopodial attachments acts independently or in tandem towards reduced invasive behavior of SW-DKK3 cells needs to be studied further.

### FOXO1 as a potential DKK3 target to effect redifferentiation

**Fig. 5:**
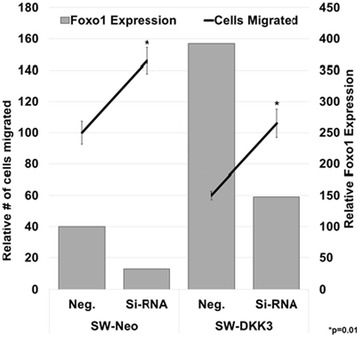
FOXO1 silencing releases DKK3-mediated block of cell migration. Cells were treated with scrambled negative siRNA (Neg.) or *FOXO1* siRNA (Si-RNA) for 24 h, trypsinized, and allowed to migrate for 4 h through modified Boyden chamber. Migrated cells were fixed, stained, and counted manually. Total number of SW-Neo cells treated with scrambled siRNA normalized to 100%, and relative change in migration of SW-Neo and SW-DKK3 cells treated with FOXO1 siRNA is shown in overlapping line graph on left Y-axis. Relative FOXO1 expression in SW-Neo and SW-DKK3 cells treated with *FOXO1* siRNA, normalized to FOXO1 expression in SW-Neo cells treated with scrambled siRNA set at 100 shown as bars on right Y-axis

Towards understanding the potential transcriptional modulation of cell adhesion and motility by DKK3 over-expression, we compared global difference in the expression pattern of 84 transcription factors using an expanded transcription array. Relative expression of 3 transcription factors, *ID1*, *JUN,* and *FOXO1*, consistently demonstrated >4-fold difference in expression between SW-DKK3 and SW-Neo/SW-13 cells (Additional file 1: Figure S8 A&B). Transcription factors *ID1* and *JUN* have been shown to mediate a variety of phenotypic effects, including apoptosis via DKK3 signaling, in multiple cancers (44, 45). DKK3-stifled invasive behavior independent of loss of viability observed in SW-13 cells prompted us to investigate a potentially novel role for FOXO1 transcription factor in DKK3-promoted redifferentiation of ACCs. Increased expression of *FOXO1* in SW-DKK3 cells was confirmed by qRT-PCR (Additional file 1: Figure S8C). Using siRNA, we transiently silenced *FOXO1* expression in SW-DKK3 and control SW-Neo cells (Additional file 1: Figure S9A&B) and assessed the effect of silencing on cell motility. Irrespective of DKK3 expression (Fig. 3b), both cell types showed an increase in migratory potential upon FOXO1 silencing (Fig. 5). The magnitude of relief in migratory inhibition was found to be more pronounced in SW-DKK3 cells (45% increase in motility with 43% FOXO1 suppression) than in SW-Neo cells (30% increase in motility with 66% FOXO1 suppression; Additional file 1: Figure S10). These results clearly suggest a role for FOXO1 in mediating DKK3-promoted redifferentiation and/or anti-invasive signaling in SW-13 ACC cells.

## Discussion

DKK3 expression is down-regulated in many human cancers, including that of the thyroid, lung, prostate, colon, breast, and liver [32, 33, 36, 43], but its regulation in ACC is unclear. In this study, we utilized comprehensive genetic, epigenetic, and functional approaches to identify and characterize a potential tumor suppressor role for DKK3 in adrenal carcinogenesis. Our study showed a significant decrease in DKK3 expression in 70% (25/37) of ACCs, strongly suggesting a tumor suppressor role for DKK3 in human adrenal tissue. Whether the observed silencing in malignant samples represents an earlier dedifferentiation or a later malignancy-promoting event needs to be determined. Despite the relatively small cohort size, this study did not find an association between DKK3 silencing and prognosis, unlike in gastric cancer [35]. Of note, the majority of this cohort of ACCs was previously shown not to harbor mutations in *DKK3* or *FOXO1* genes while <10% carried beta-catenin mutations [24].

Epigenetic modifications, including promoter methylation and chromatin condensation, have been proposed as major DKK3 silencing mechanisms in a variety of tumors [43]. This study also supports a role for promoter hypermethylation in DKK3 silencing in ACCs. Interestingly, DKK3 expression was also significantly decreased in many samples with intermediate methylation (48%), suggesting that even intermediate levels of methylation may be adequate to silence DKK3 expression. Whether the *DKK3* promoter methylation observed in this study is a component of the global methylation changes observed in ACCs [9, 40] or a specific *DKK3* gene-targeted event needs to be clarified. A large proportion of the ACC study cohort with non-methylated promoters but with reduced DKK3 expression led us to seek alternate mechanisms for DKK3 down-regulation in ACC. In light of recent findings that gene copy number variations may contribute to adrenocortical carcinogenesis [8, 24], we analyzed a portion of our samples for *DKK3* gene copy number variations. The majority of samples identified with *DKK3* copy loss also had significantly reduced DKK3 expression. Only a handful of these samples had concurrent promoter methylation, indicating a possible independent role for gene copy loss in causing DKK3 down-regulation in ACC. One ACC sample with 6 copies of *DKK3* and a hypermethylated promoter had significantly reduced expression of DKK3, suggesting that copy number variations may occur earlier in ACC oncogenesis than gene-specific methylation events.

Statistical correlation to patient characteristics and outcomes did not reveal any prognostic association of reduced DKK3 expression in ACC patients, although reduced DKK3 expression was found to trend non-significantly toward female gender. This study did not reveal a relationship between DKK3 expression and aldosterone biosynthesis, as reported earlier [25]. In addition, no significant correlation was observed in our tumor cohort between DKK3 expression, metastasis, and tumor grade.

We used functional approaches to characterize the effects of DKK3 on human ACC cells. Silencing of DKK3 in SW13, a human ACC cell line with intact and inductile WNT signaling and endogenously expresses DKK3, did not affect growth or viability of cells but resulted in reduced clonogenic growth and increased motility, consistent with a tumor suppressor role for DKK3 [31, 41]. In contrast, exogenous addition of DKK3 to SW-13 cells resulted in increased motility, suggesting distinct roles for intracellular and secreted DKK3s. This observation is consistent with recent suggestions that DKK3 potentially has distinct intracellular signaling partners independent of canonical WNT-β-catenin circuitry [26]. Overall, intracellular DKK3 appears to confer a more differentiated phenotype to SW-13 cells. Whether the observed DKK3-promoted more differentiated phenotype is through (a) reactivation of the proposed adrenocortical differentiation pathway [14], (b) blocking of malignancy signaling networks, or (c) activation of a novel redifferentiation pathway needs to be clarified.

Light microscopic analysis revealed drastic changes in organization of cell outgrowths on the edges of slow-moving SW-DKK3 cells. While parental SW-13 and SW-Neo cells produced an overwhelming number of dynamic filopodia that confer polarity and promote directional movement (41–43), SW-DKK3 cells showed predominantly lobopodia, indicative of multipolar spreading and hence arrested motility [44–46]. Although DKK3 has previously been shown to influence migratory and invasive phenotypes in multiple cancer cell types, an association of cell surface modifications that can impact cell mobility has not been shown. The mechanism(s) that elicit the observed changes in cell extension repertoire need to be investigated further. The association of the dedifferentiated phenotype and loss of DKK3 expression in ACC, combined with the re-acquisition of a relatively more differentiated phenotype in SW-13 ACC cells overexpressing DKK3, suggest a global differentiation role for DKK3 in adrenal cortex and the possibility that DKK3 could serve as a re-differentiation therapeutic target.

To explore potential pathways involved in eliciting the observed DKK3-promoted redifferentiated phenotype of ACC cells, we compared the expression pattern of 84 human transcription factors. Of the 3 transcription factors found to be over-expressed in SW-DKK3 cells (*ID1, JUN and FOXO1*), *FOXO1* secured our immediate attention for 3 primary reasons: (1) *FOXO1* is known to promote functional differentiation of myofibroblasts [47], (2) *FOXO1* inhibits osteosarcoma malignancy via WNT inhibition [48], and (3) *FOXO1* transcription has been suggested in response to steroid hormones [49]. We hypothesized that intracellular DKK3 promotes cellular differentiation signaling encompassing cellular spreading and stifled motility, at least in part, via *FOXO1* up-regulation. *FOXO1* RNAi silencing resulted in partial reversal of the motility suppression, suggesting that *FOXO1* may indeed play a role in DKK3-promoted redifferentiation of ACC cells. Based on the inverse relationship observed in ACC tissue between DKK3 and beta-catenin expression, it can be assumed that DKK3/FOXO1 regulation of malignant behavior of SW-13 cells is mediated through beta-catenin signaling. However, the precise DKK3-FOXO1 signaling circuitry in the context of adrenocortical differentiation needs to be investigated further.

## Conclusions

In conclusion, we demonstrate for the first time that DKK3 expression is frequently reduced in ACC, potentially contributing to adrenal dedifferentiation and/or progression of malignancy. Further, we identified *FOXO1* as a downstream effector of *DKK3* that may play a role in blocking adrenocortical dedifferentiation. These results suggest a potential for developing novel redifferentiation-focused pharmaceuticals that could allow successful treatment of ACCs when used in concert with existing treatment regimens.

## Additional file

Additional file 1: Figure S1-S10. Supplementary Figures S1-S10. (PPTX 34931 kb)

## Abbreviations

ACC: Adrenocortical carcinoma
DKK3: Dickkopf-related protein 3
FBS: Fetal bovine serum
FF: Flash-frozen
FFPE: Formalin-fixed, paraffin-embedded
FITC: Fluorescein isothiocyanate
FOXO1: Forkhead Box Protein O1
H&E: Hematoxylin & eosin
RPLP0: Ribosomal protein lateral stalk subunit P0
RPPH1: Ribonuclease P RNA component H1
TR: Texas Red
