# Supplementary material for: A novel FOXO1-mediated dedifferentiation blocking role for DKK3 in adrenocortical carcinogenesis
Source: BMC Cancer. 2017 Mar 1;17:164. doi: 10.1186/s12885-017-3152-5 (PMC5333434; doi:10.1186/s12885-017-3152-5)
Supplement: Additional file 1: Figure S1-S10. — Supplementary Figures S1-S10. (PPTX 34931 kb) [file 12885_2017_3152_MOESM1_ESM.pptx]

## Slide 1
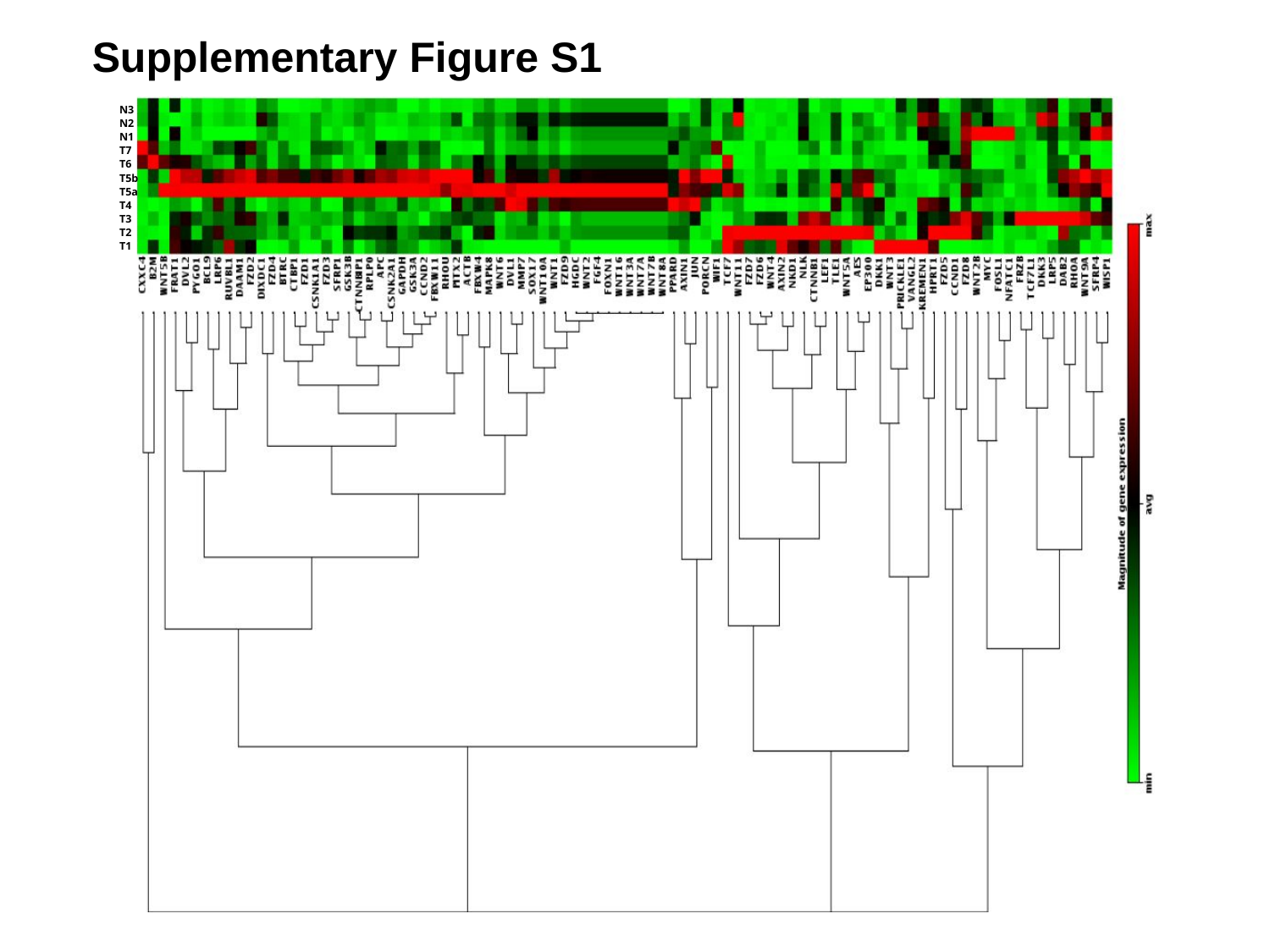

Supplementary Figure S1
N3
N2
N1
T7
T6
T5b
T5a
T4
T3
T2
T1

## Slide 2
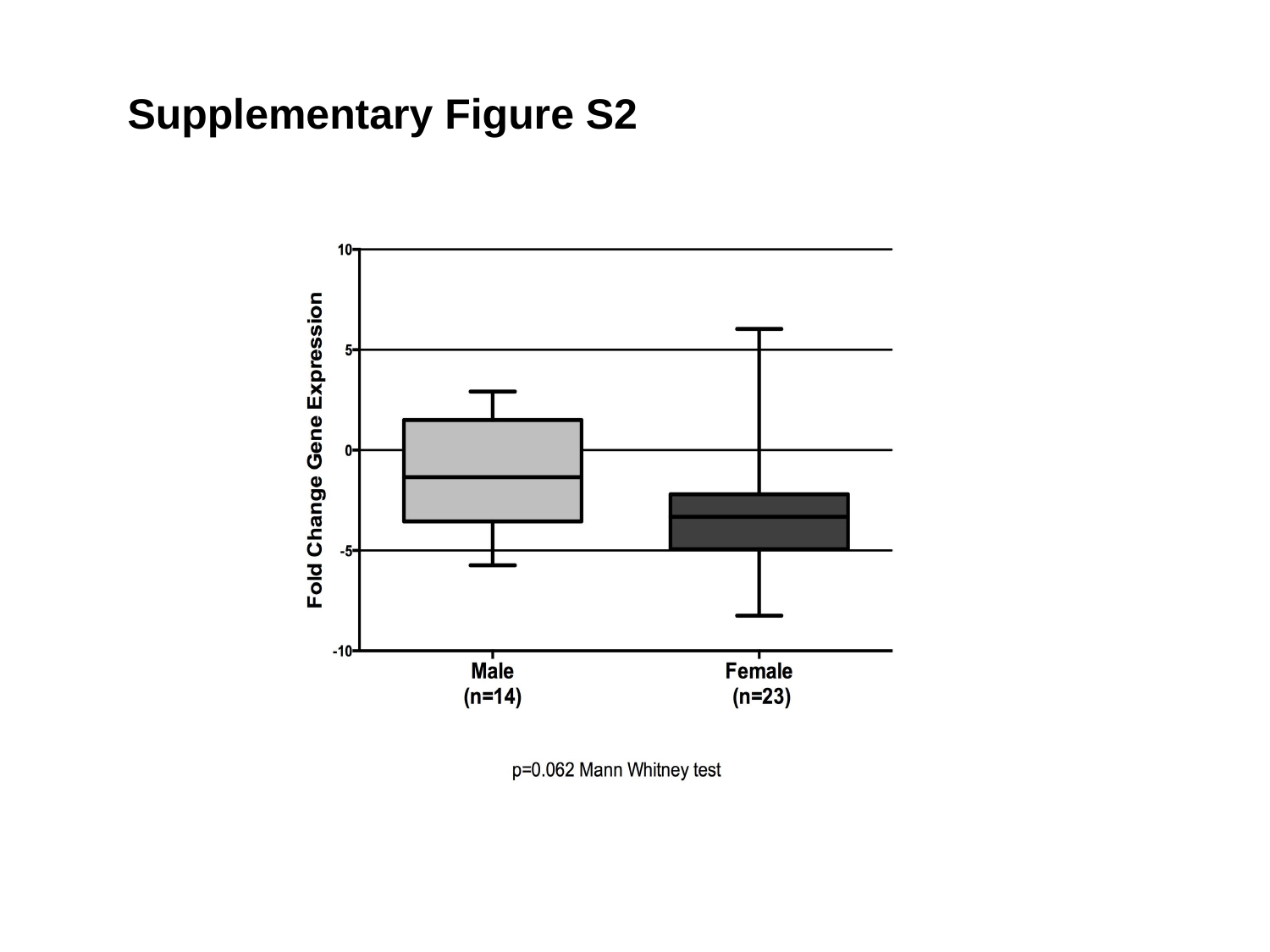

Supplementary Figure S2

## Slide 3
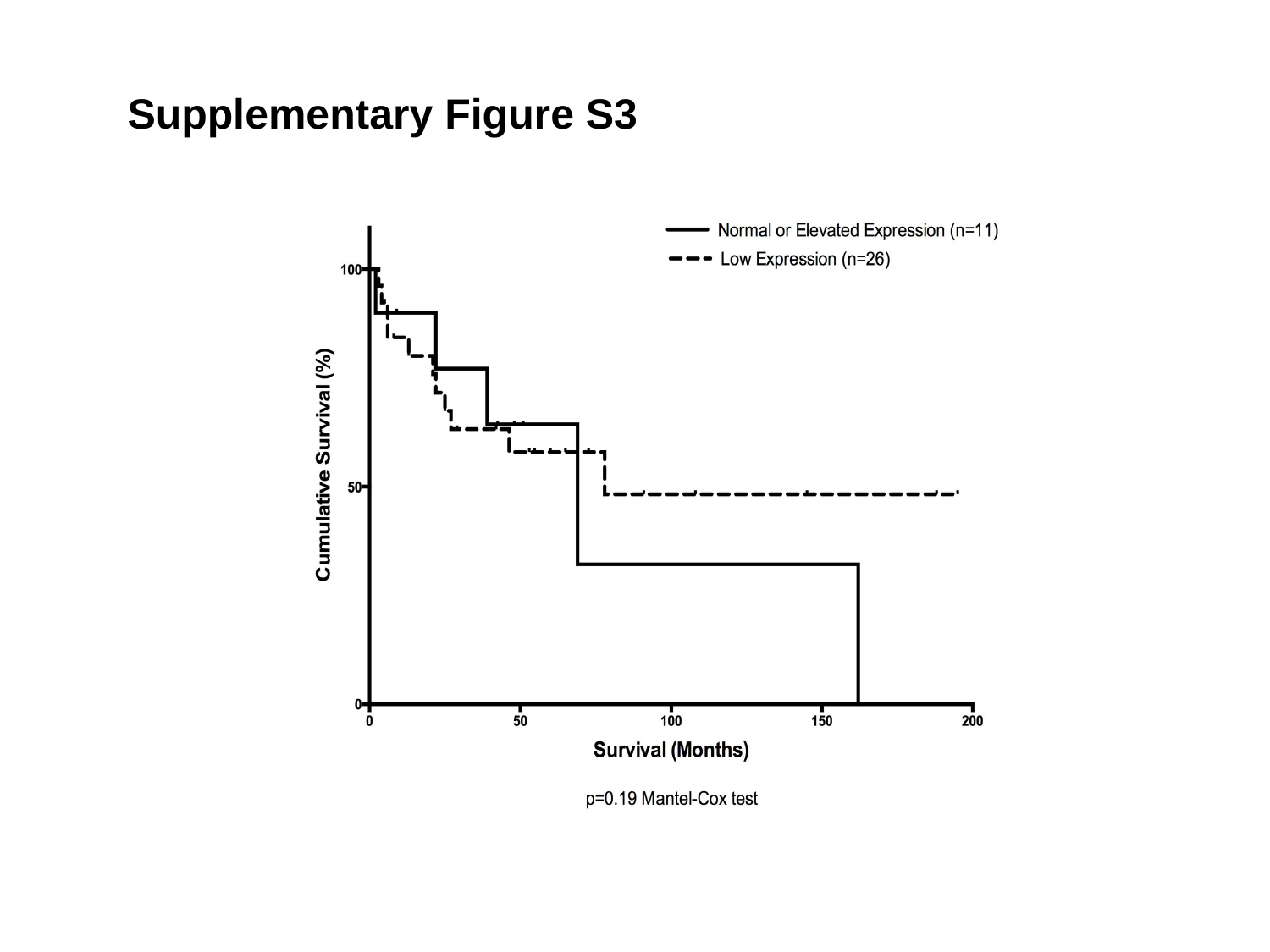

Supplementary Figure S3

## Slide 4
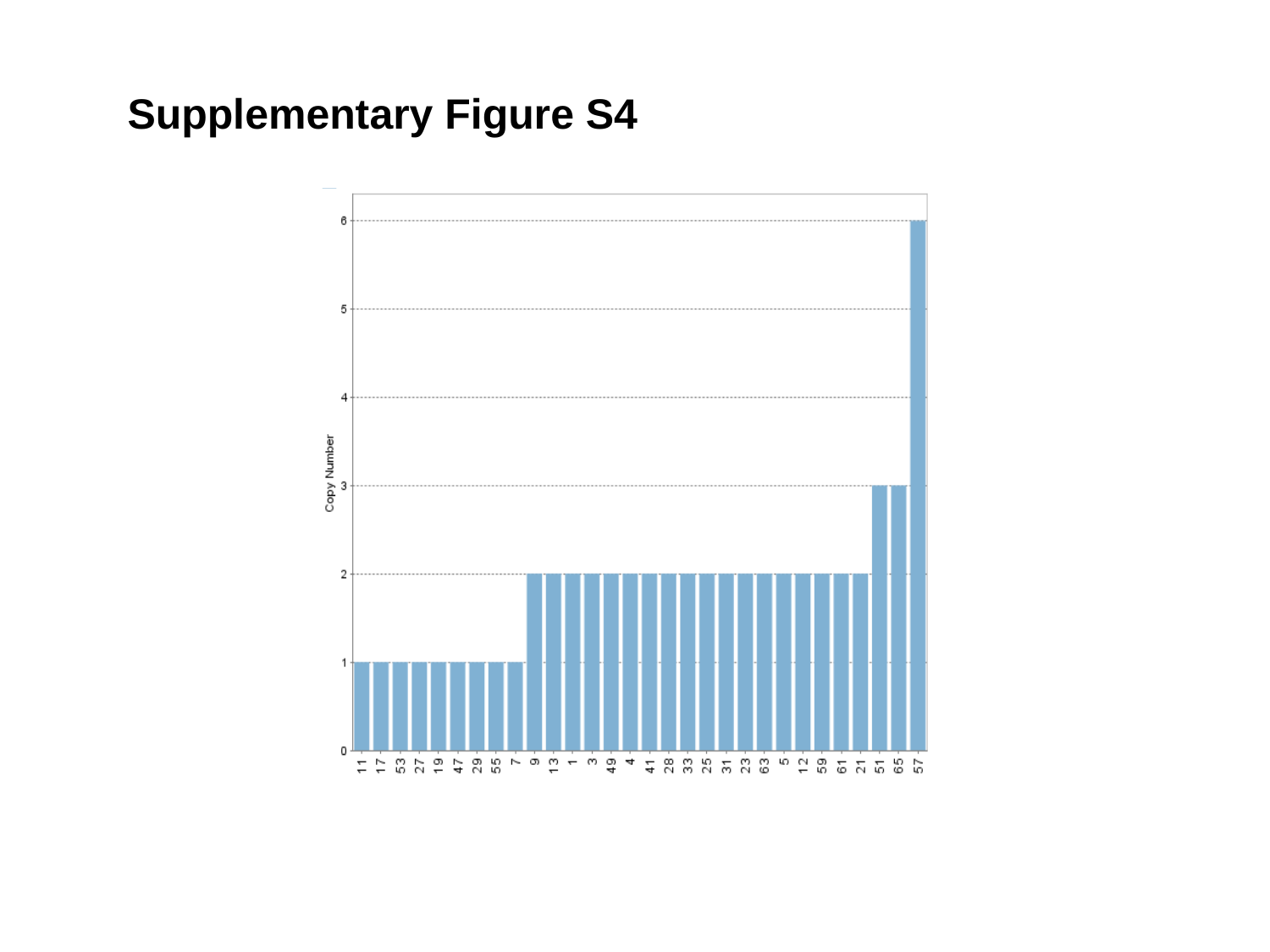

Supplementary Figure S4

## Slide 5
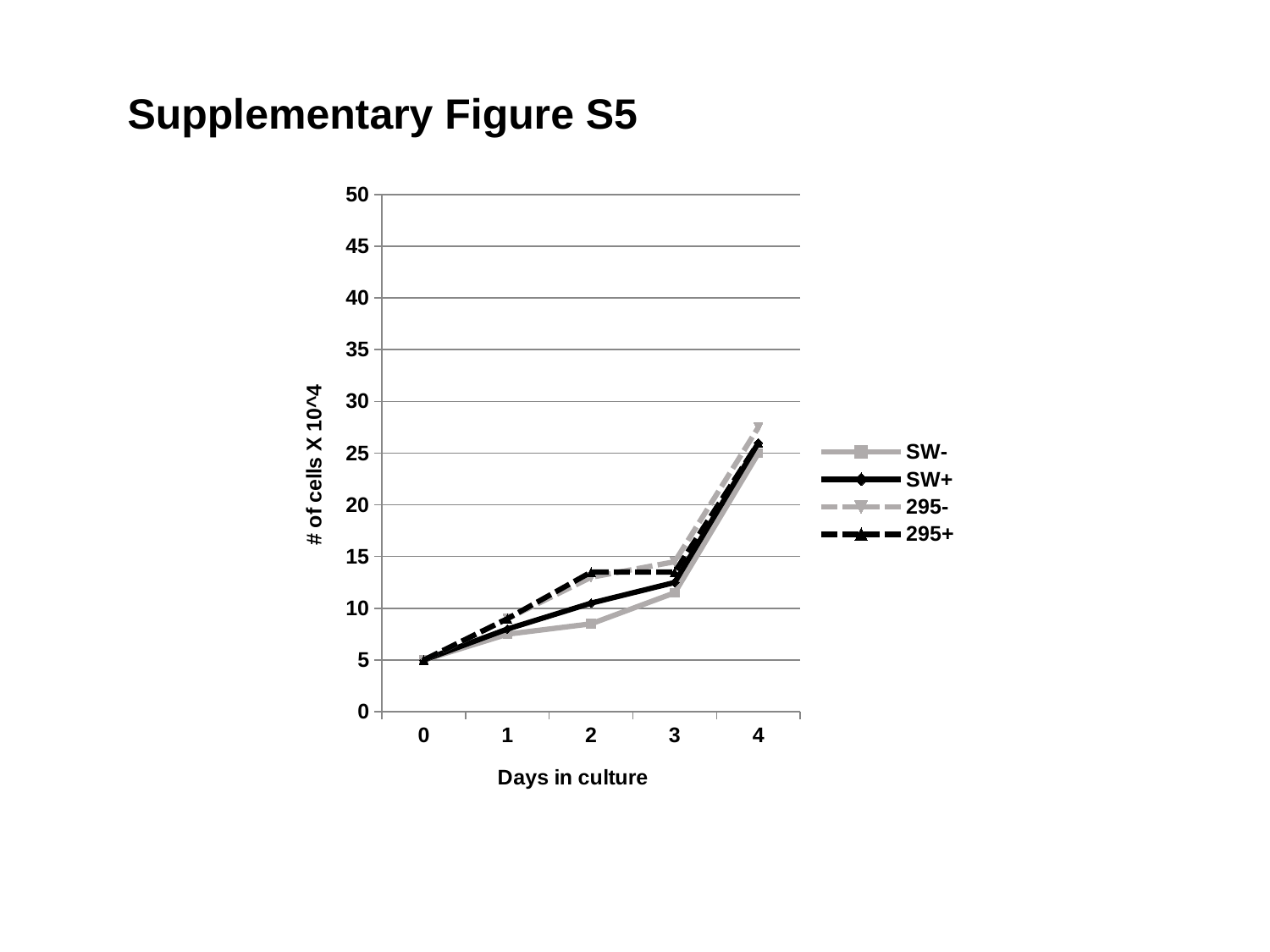

Supplementary Figure S5
### Chart
| Category | SW- | SW+ | 295- | 295+ |
|---|---|---|---|---|
| 0 | 5.0 | 5.0 | 5.0 | 5.0 |
| 1 | 7.5 | 8.0 | 9.0 | 9.0 |
| 2 | 8.5 | 10.5 | 13.0 | 13.5 |
| 3 | 11.5 | 12.5 | 14.5 | 13.5 |
| 4 | 25.0 | 26.0 | 27.5 | 26.0 |

## Slide 6
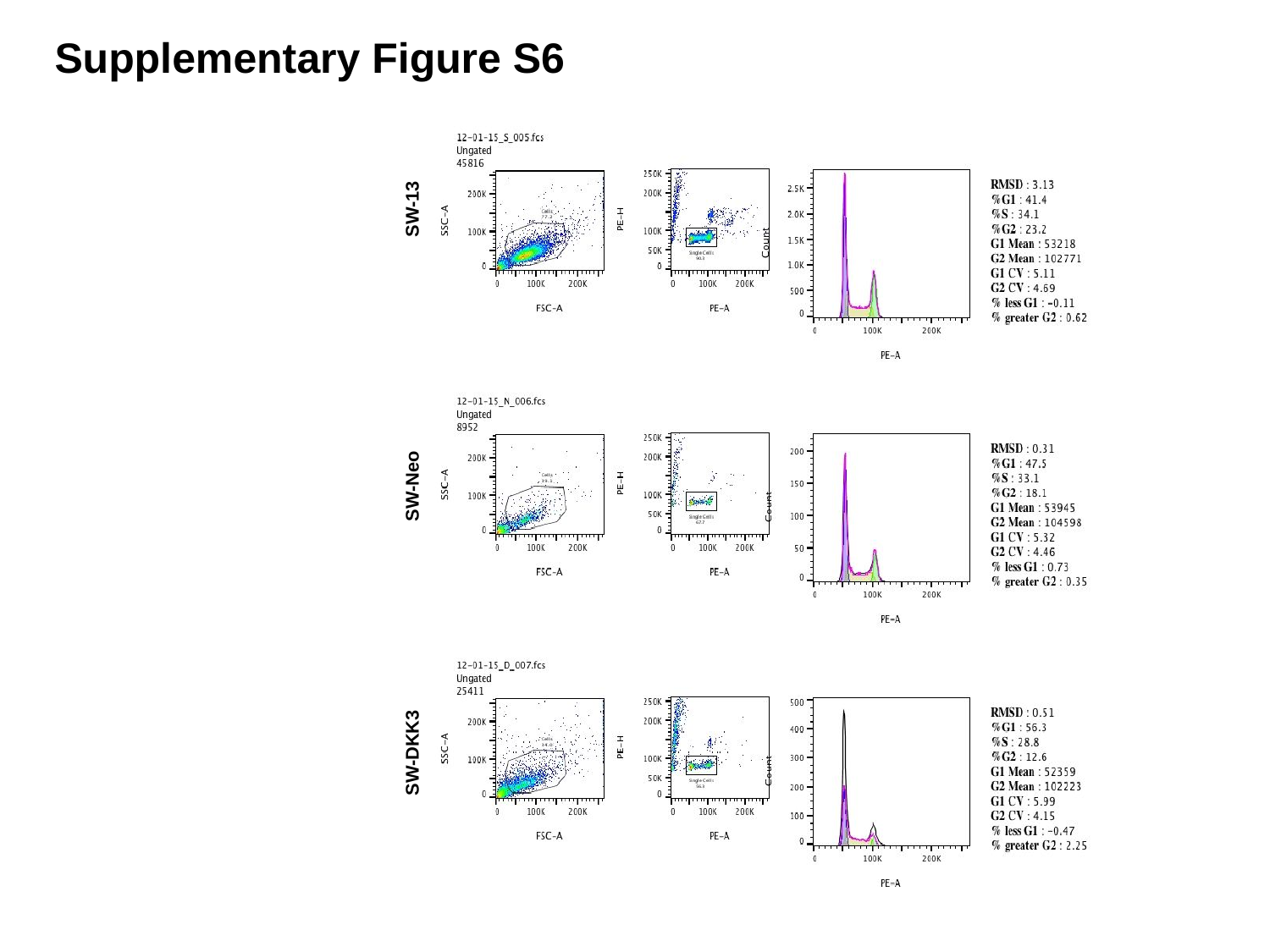

Supplementary Figure S6
SW-DKK3	 SW-Neo	 SW-13

## Slide 7
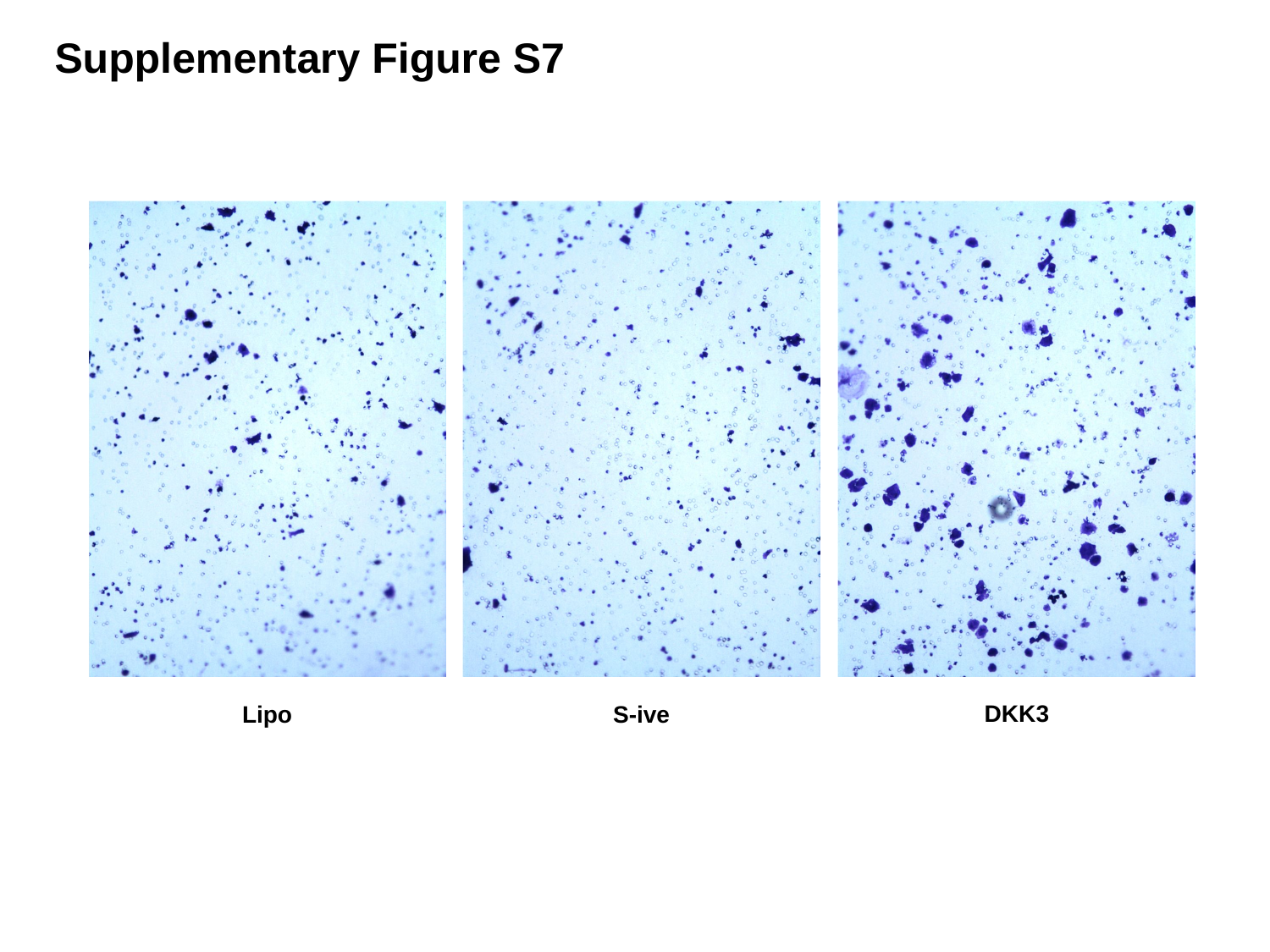

Supplementary Figure S7
DKK3
Lipo
S-ive

## Slide 8
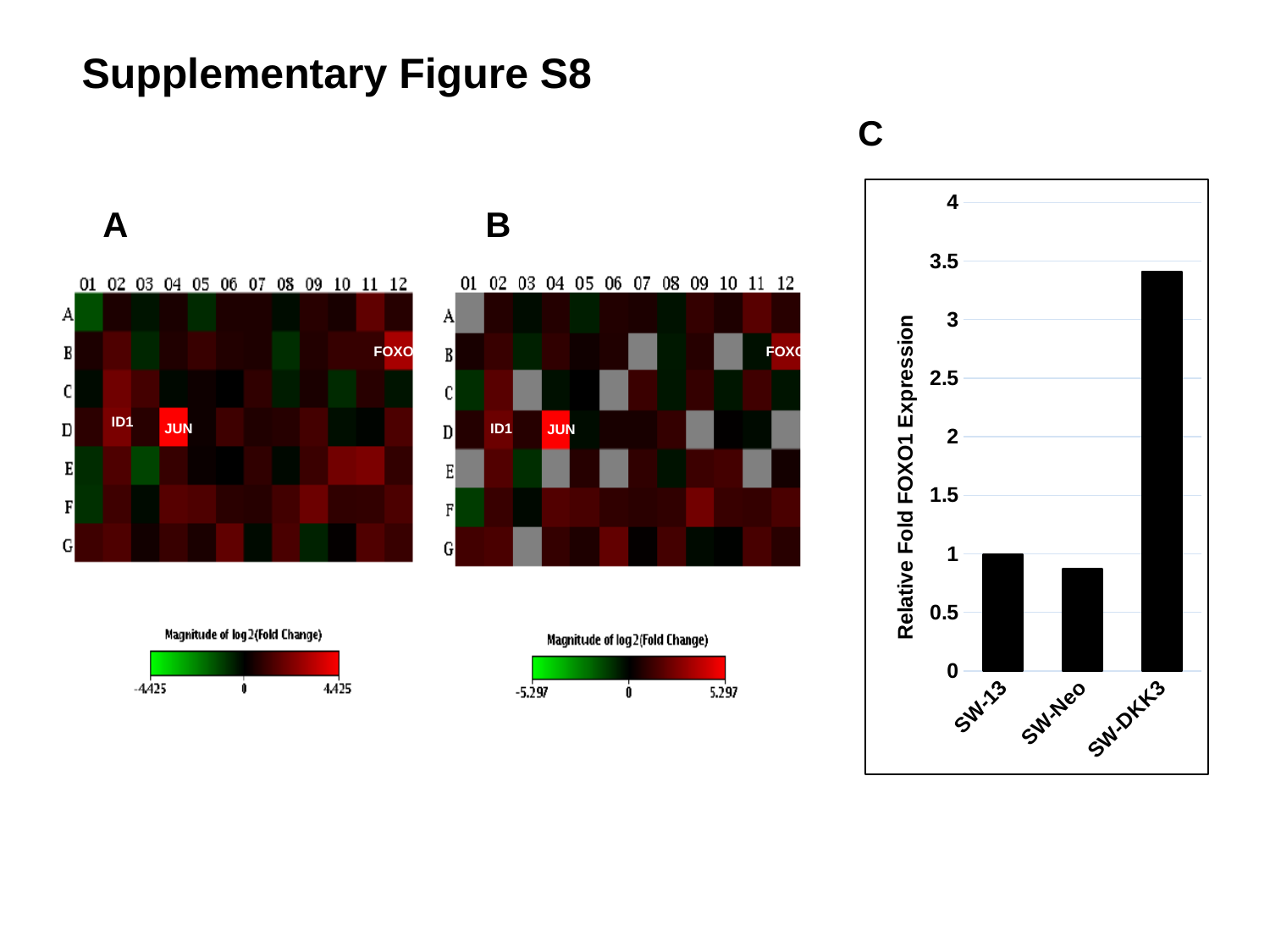

Supplementary Figure S8
C
### Chart
| Category | Foxo1 |
|---|---|
| SW-13 | 1.0 |
| SW-Neo | 0.87 |
| SW-DKK3 | 3.41 |A
B
FOXO1
FOXO1
ID1
JUN
ID1
JUN

## Slide 9
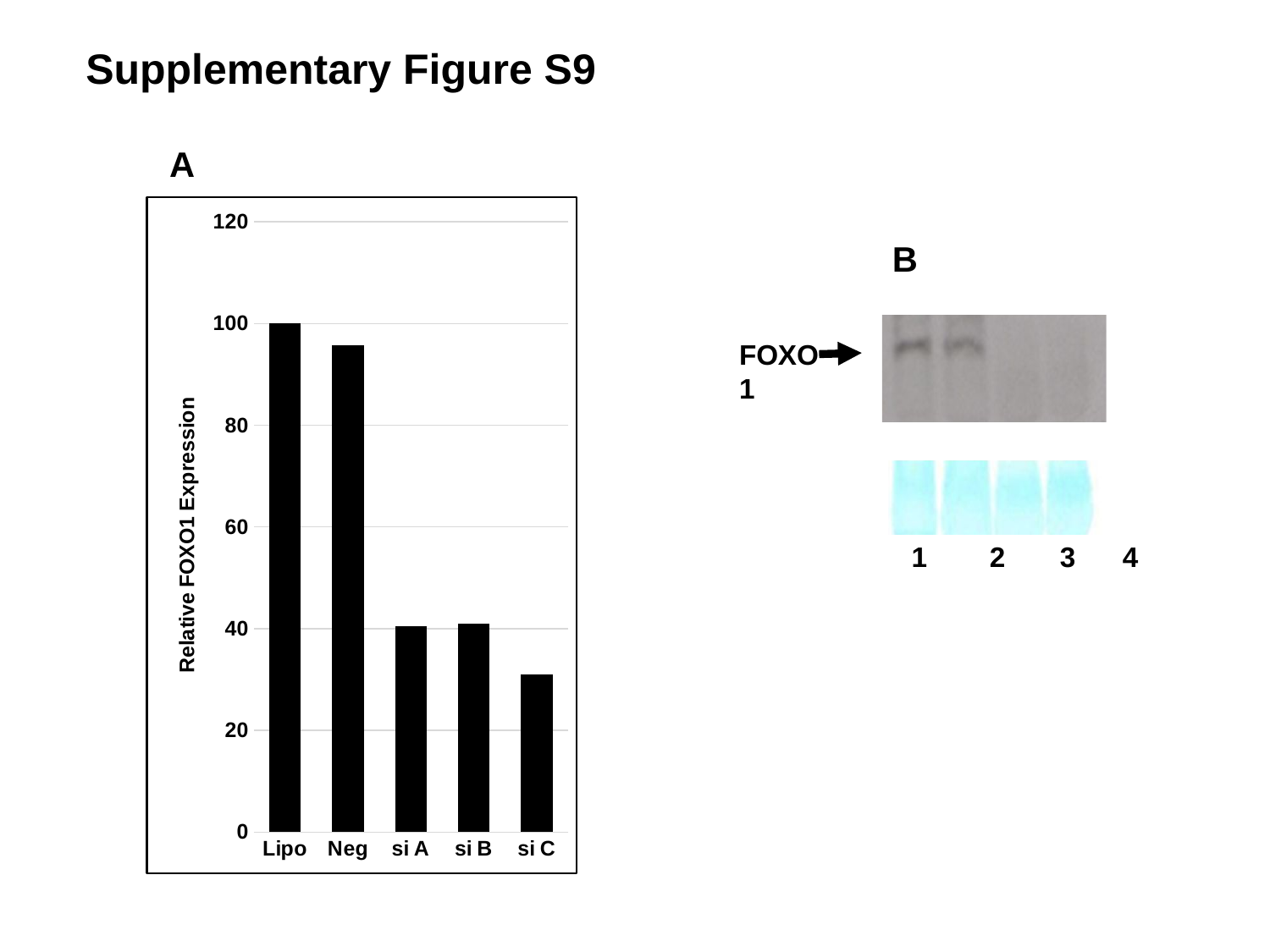

Supplementary Figure S9
A
### Chart
| Category | |
|---|---|
| Lipo | 100.0 |
| Neg | 95.65757554322808 |
| si A | 40.563402354021 |
| si B | 40.92817698146658 |
| si C | 30.95805021288289 |B
FOXO1
1 2 3 4

## Slide 10
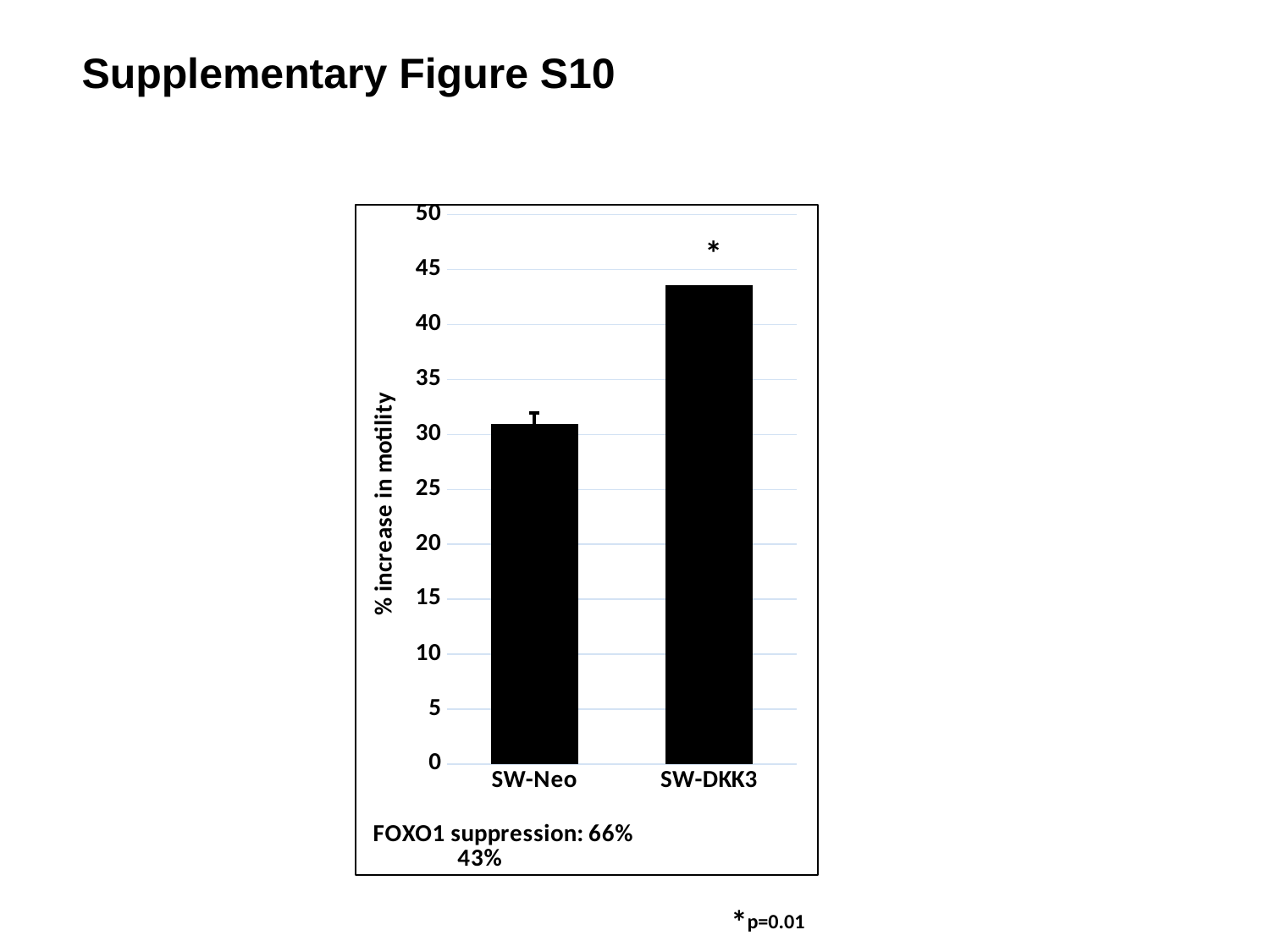

Supplementary Figure S10
### Chart
| Category | |
|---|---|
| SW-Neo | 30.92369477911646 |
| SW-DKK3 | 43.61111111111111 |*
*p=0.01
